# DLGAP4 acts as an effective prognostic predictor for hepatocellular carcinoma and is closely related to tumour progression

**DOI:** 10.1038/s41598-022-23837-y

**Published:** 2022-11-17

**Authors:** Cairong Dong, Shenglan Huang, Liang Sun, Jinping Yao, Jinlong Yan, Xiangbao Yin

**Affiliations:** 1 Department of General Surgery, The Second Affiliated Hospital of Nanchang University, Nanchang, Jiangxi Province People’s Republic of China; 2 Department of Oncology, The Second Affiliated Hospital of Nanchang University, Nanchang, Jiangxi Province People’s Republic of China; 3 Department of Endocrinology Department, The Fourth Affiliated Hospital of Nanchang University, Nanchang, Jiangxi Province People’s Republic of China

**Keywords:** Cancer, Genetics

## Abstract

Disc large associated protein 4 (DLGAP4) plays an important role in neurological diseases, but the role and mechanism of DLGAP4 in hepatocellular carcinoma (HCC) remain unclear. In this study, the prognostic effect of DLGAP4 on HCC patients was investigated by means of bioinformatics. The correlation of DLGAP4 expression with the prognosis of HCC patients was evaluated by TCGA data analysis, and the correlation between DLGAP4 expression and the clinical characteristics of HCC patients was evaluated by the Wilcoxon signed rank test and logistic regression analysis. Kaplan‒Meier and Cox regression methods were used to assess the effect of DLGAP4 expression level on overall survival, and nomograms were used to illustrate the correlation between DLGAP4 gene expression and HCC risk. The genes related to DLGAP4 in HCC were screened, and GO/KEGG enrichment analysis was performed. Furthermore, in vitro and in vivo experiments were conducted to detect the effect of DLGAP4 expression on the proliferation, migration and metastasis of HCC cells. We also examined the effect of DLGAP4 expression on enriched pathway proteins to explore the possible mechanism. The expression levels of DLGAP4 were significantly higher in HCC cell lines and tissue samples than in normal liver cell lines and tissues. The expression of DLGAP4 was significantly associated with clinical characteristics. Survival analysis showed that high expression of DLGAP4 was associated with a poor prognosis in HCC. Multivariate analysis showed that high expression of DLGAP4 was an independent risk factor affecting the overall survival rate in HCC patients. By means of ROC curve analysis and nomograms, we determined the value of DLGAP4 expression in the diagnosis and prognosis evaluation of HCC. GO/KEGG enrichment analysis showed that the PPAR signalling pathway was differentially enriched in patients with high expression of DLGAP4. According to in vitro and in vivo experiments, DLGAP4 knockdown inhibited the proliferation and metastasis of HCC cells and decreased the expression of PPARβ/δ protein. In contrast, overexpression of DLGAP4 promoted the proliferation and metastasis of HCC cell, and increased the expression of PPARβ/δ protein.In contrast, overexpression of DLGAP4 promoted the proliferation and metastasis of HCC cells and increased the expression of PPARβ/δ protein. The results show a close correlation between DLGAP4 expression and clinicopathological features of HCC, and DLGAP4 can be used as a prediction biomarker of HCC.

## Introduction

Liver cancer is one of the most common cancers in the world, and more than 90% of cases are hepatocellular carcinoma (HCC)^1^. Both morbidity and mortality associated with HCC have increased substantially over the past few decades^2^. The main treatment for HCC is comprehensive therapy^3^. However, the prognosis of HCC is very poor, with an overall 5-year survival rate of 10–20%^4^. Due to the lack of specific early markers of HCC, most patients are already in the advanced stage at the time of initial diagnosis^5^. In precision medicine, key genes that drive cancer can be considered therapeutic targets. Discovery of new therapeutic targets will help improve outcomes for patients with HCC. Therefore, there is an urgent need to find new HCC markers.

The discs large associated protein (DLGAP) family has five members, DLGAP1-5, which are distributed on different chromosomes and produce transcript variants of different lengths^6^. DLGAPs are highly conserved across species, and the homology between members varies from 26 to 48%^6^. DLGAP4 is an important member of DLGAPs, and a large number of studies have shown that the function of the DLGAP4 gene is related to a variety of neurological diseases, including schizophrenia^7^, trichotillomania^8^, obsessive–compulsive disorder^9^, and cerebellar ataxia^10^. However, to date, little research has been done on the role of DLGAP4 in HCC. Therefore, in this study, we explored the biological function and possible mechanism of DLGAP4 in HCC.

Peroxisome proliferator-activated receptor (PPARs) are ligand-activated transcription factors, and this family includes three subtypes: PPARα, PPARβ/δ and PPARγ. Their expression patterns are often tissue specific, their responses vary by ligand, and their activities are regulated by ligand-induced conformational changes through differential recruitment of cofactors^11^. Among the subtypes, PPARβ/δ is the most widely expressed in human tissues and is abundantly present in the skin, intestine and liver^12^. In cancer, PPARβ/δ regulates tumour cell proliferation, differentiation, invasion, metastasis and other biological behaviours^13^. The expression of PPARβ/δ in human non-small cell lung cancer tissue is significantly higher than that in normal lung tissue, and the activation of PPARβ/δ can promote the proliferation and viability of lung cancer cell lines, whereas the interference of PPARβ/δ expression can increase the apoptosis of cancer cells^14,15^. PPARβ/δ plays a key role in the occurrence and development of HCC. Previous studies have shown that PPARβ/δ activation promotes the proliferation of human hepatoma cells by upregulating COX-2 and prostaglandin E2 production^16^. SGK1 can promote HCC progression, and PPARβ/δ inactivation inhibits the cancer-promoting effect of SGK1^17^. Therefore, PPARβ/δ is an important signalling pathway mediating HCC progression. However, the relationship between DLGAP4 regulation of HCC biological behaviour and PPARβ/δ is not clear.

In recent years, a large number of database platforms have emerged, and researchers can easily use these data platforms to conduct cancer bioinformatics research so that markers can be easily screened. To this end, to clarify the biological function of the DLGAP4 gene in HCC, this study aimed to evaluate the role of DLGAP4 gene expression in HCC by bioinformatics analysis of clinical features and survival information in The Cancer Genome Atlas (TCGA). We also performed in vitro and in vivo experiments to investigate the effect of DLGAP4 expression on HCC cell proliferation and invasion and related key signalling pathways.

## Materials and methods

### Patient datasets

mRNA expression data (371 samples, workflow type: HTSeq-FPKM) and clinical information were downloaded from the TCGA database (https://cancergenome.nih.gov). Exclusion criteria: (1) No DLGAP4 gene expression data (2) The patient’s clinical data were incomplete. A total of 371 eligible patients were included in this study. We obtained the gene amplification and mutation status of DLGAP4 through cBioPortal for Cancer Genomics (http://www.cbioportal.org/). The GSCA database (http://bioinfo.life.hust.edu.cn/GSCA/#/) was used to analyse the correlation between DLGAP4 differential expression and methylation in HCC, and it was also used to analyse DLGAP4 copy number variation in HCC. The Human Protein Atlas is an interactive web-based database (https://www.proteinatlas.org), containing RNA and protein expression profiles of more than 90% of putative protein-coding genes, including more than 13 million high-resolution images^18^. The expression of DLGAP4 in HCC was detected at the protein level using this database to verify its differential expression in tumour and normal tissues.

### Survival analysis

The Kaplan‒Meier plotter (http://kmplot.com/analysis/)^19^ was used to estimate the correlation between DLGAP4 expression and the survival rate of HCC patients, and the hazard ratio (HR) and log-rank p value of the 95% confidence interval were calculated.

### TIMER2.0

TIMER2.0 (http://timer.comp-genomics.org/) is a database related to tumour immunity^20^. It is a convenient and accurate online analysis tool to explore the gene expression levels in normal and tumour tissues in TCGA datasets to infer the abundance of tumour-infiltrating immune cells from gene expression profiles and to assess their clinical influence. The database contains three parts: immune Association, Immune Estimation and Cancer Exploration. In this study, the Gene_DE plate of Cancer Exploration was selected to obtain the differential expression of DLGAP4 in tumour tissues and normal tissues in different tumour types.

### Establishment and evaluation of the nomograms for HCC survival prediction

In this study, we selected all independent clinicopathological prognostic factors in the Cox regression analysis and constructed contingency tables (nomograms) to predict 1-, 3-, and 5-year overall survival (OS) in patients with HCC. The accuracy of the line graph can be verified by comparing the actual probability observed with the calibration curve to the predicted probability of the line graph^21^. Overlapping reference lines indicate that the model is accurate. The TCGA and International Cancer Genome Consortium (ICGC) (https://dcc.icgc.org/) databases were queried to conduct receiver operating characteristic (ROC) curve analysis to compare the prediction accuracy of the nomogram versus various clinicopathological prognostic factors.

### Sample collection

Twenty pairs of HCC patient specimens and adjacent normal tissues from patients who were admitted to the Department of Hepatobiliary Surgery of our hospital and underwent surgical treatment were collected. Specimens were stored at − 80 °C immediately after acquisition. This study was approved by the Ethics Committee of the Second Affiliated Hospital of Nanchang University. All samples were used in accordance with the statutes of the Declaration of Helsinki.

### Cell culture

The human normal liver cell line LO2 and human HCC cell lines (HepG2, HCCLM3, MHCC97H) were obtained from the Cell Bank of the Chinese Academy of Sciences (Shanghai, China). The cells were cultured at 37 °C and 5% CO2 in a humidified atmosphere incubator. The cells were cultured in DMEM (Doctor DE Biological, Wuhan, China) supplemented with 10% foetal bovine serum (FBS; Gibco) and antibiotics (100 units/ml penicillin and 100 µg/ml streptomycin). The liquid was changed every 2 days, and the growth of the cells was observed under an inverted microscope.

### Plasmid construction and lentiviral transfection

A plasmid containing DLGAP4-interfering RNA (shRNA) was constructed by Hanbio Tech (Shanghai, China) and packaged into lentivirus particles. The sequences were as follows: DLGAP4-targeted plasmid (Gene ID: 22839) interfering sequence sense strand: GCUACUUCAUGCACGCCUAT, antisense strand: UAGGCGUGCAUGAAGUAGCTT; shRNA negative control (shNC) sequence: sense strand: UUCUCCGAACGUGUCACGUTT, antisense strand: ACGUGACACGUUCGGAGAATT. The successfully constructed interference and control viruses were transfected into HepG2 and HCCLM3 HCC cell lines. Stable DLGAP4 knockdown cell lines were constructed for functional experiments. The DLGAP4 overexpression plasmid (transcript number: NM_001365621.2) was constructed by Hanbio Tech (Shanghai, China). DLGAP4-flag or Vector-flag was transferred into the MHCC97h HCC cell line. The experimental procedures were described previously^22^.

### Quantitative real-time polymerase chain reaction (PCR) of cell lines

Total RNA was extracted from cells using TRIzol (Solarbio, Beijing, CHN). PCR amplification was then performed using the TagMan mRNA Reverse Transcription Kit (Doctor DE Biological, Wuhan, China) (reverse transcription reaction system: 16 °C for 30 min, 42 °C for 30 min, 85 °C for 5 min). Subsequently, using the cDNA as a template, a 20 µl system was configured for PCRs using a Takara qPCR kit (Wuhan Boster, Wuhan, China). Finally, quantitative real-time PCR (qRT PCR) analysis was performed with SYBR^®^ Green (Roche, Basel, Switzerland). The CT values of DLGAP4 were normalized to those of GAPDH by subtracting the mean CT value of each sample. The relative quantification (RQ) value of DLGAP4 in each cell was calculated using the 2^−ΔΔct^ method. The quantitative real-time PCR primer sequences were as follows:

- DLGAP4 forward primer 5′-GCTGTCTCTTTGTCTCTGCCC-3′
- DLGAP4 reverse primer 5′-TGGAAGGTGTTCTCAAGGGG-3′
- GAPDH forward primer 5′-GGAGCGAGATCCCTCCAAAAT-3′
- GAPDH reverse primer 5′-GGCTGTTGTCATACTTCTCATGG-3′

### Immunochemistry

The ethics and informed consent involved in the human tissue experiments are detailed in “Ethical approval” section. Tissues were sectioned and dewaxed. Subsequently, sections were incubated with rabbit anti-DLGAP4 (1:100, Bioss, China) overnight at 4 °C. The sections were then washed three times with phosphate buffered saline. After 30 min of incubation with goat anti-rabbit IgG (1:500, Bioss, China), all sections were stained with 3,3′-diaminobenzidine (DAB). Then, the sections were washed and sealed, and the positive cells labelled by DLGAP4 were observed under an inverted fluorescence microscope.

### CCK-8 cell proliferation assay

Cell viability was determined with a CCK-8 assay. Hepatoma cells were seeded into 96-well plates at 4 × 10^3^ cells per well and incubated for 0 h, 24 h, 48 h, and 72 h. Then, 10 µl of CCK-8 reagent (Doctor DE Biological, Wuhan, China) was added to each well. After 1 h of incubation at 37 °C in the dark, the absorbance was measured at 450 nm using an iMark microplate reader (Bio-Rad). Five duplicate wells were measured for the experimental groups and control groups, and the measurements were averaged.

### EdU cell proliferation assay

The EdU Cell Proliferation Kit with Alexa Fluor 647 (Beyotime, Shanghai, CHN) was used to evaluate cell proliferation. HCC cells were incubated with 5-ethynyl-20-deoxyuridine (EdU) for 4 h and subsequently processed according to the manufacturer's instructions. The specific experimental procedures are described in^23^.

### Transwell migration assay

An 8 mm transwell chamber (Boster China) was used to detect the effect of DLGAP4 expression on the migration ability of hepatoma cells. Cells were detached from culture flasks and resuspended in serum-free DMEM. Cells were counted with a cell counter. According to the count, the cell suspension was adjusted to a concentration of 4 × 10^4^ cells/200 µl. Then, 200 µl of serum-free cell suspension was inoculated into the upper chamber, and 600 µl of DMEM complete medium containing 10% FBS was inoculated into the lower chamber as a chemoattractant. The cells were then returned to the chamber for 72 h of incubation; then, they were removed, and the unmigrated cells in the upper layer of the chamber were gently wiped off with a thin cotton swab. The inserts were fixed in 5% formaldehyde solution for 10 min. After that, each chamber was stained with 0.1% crystal violet for 10 min and then washed three times with PBS. Three random microscope fields were captured, and cells were counted for each group.

### Western blot experiments and antibodies

Tissues and cells were lysed using RIPA lysis buffer (Solarbio, Beijing, CHN) containing protease inhibitors to extract proteins. The protein samples were electrophoresed.The gel and membrane were cut to the same size according to the target protein locations indicated by the marker before trarsmembrane. Then,the samples were transferred to membranes, blocked, and incubated with antibodies. The results were analysed using ImageJ. The following antibodies were used: rabbit PPARβ/δ monoclonal antibody (1:2000, Abcam, Cambridge, MA, USA), mouse β-actin polyclonal antibody (1:1000, Boster Biological Technology, Ltd), rabbit E-cadherin polyclonal antibody (1:1000, Boster Biological Technology, Ltd), rabbit N-cadherin polyclonal antibody (1:1000, Boster Biological Technology, Ltd), rabbit DLGAP4 polyclonal antibody (1:1000, Abcam, Cambridge, MA, USA), and rabbit CyclinD1 polyclonal antibody (1:1000, Boster Biological Technology, Ltd.). Secondary lgG goat-antibody rabbit (1:10,000, Beyotime, Shanghai, China) or secondary lgG goat-antibody mouse (1:10,000, Beyotime, Shanghai, China) was used.

### In vivo experiment

The ethics involved in animal experiments are detailed in “Ethical approval” section. BALB/C nude mice aged 5–6 weeks were purchased and housed in a sterile environment with controlled temperature and light. Tumorigenicity experiment: Nude mice were randomly divided into 2 groups with 6 mice in each group. HepG2 cells stably transfected with shNC and shDLGAP4 were collected and resuspended in 100 µl PBS, and the number of cells was approximately 1 × 107. The cells were injected separately into the flank abdominal wall of nude mice. Four weeks after inoculation, all mice were euthanized after anaesthesia, and the tumours were excised, photographed, and weighed. Metastasis experiment: Nude mice were randomly divided into 2 groups with 6 mice in each group. HepG2 cells transfected with shNC and shDLGAP4 were resuspended in 100 µl PBS, with approximately 1 × 107 cells in each group. HepG2 cell suspensions stably transfected with shNC and shDLGAP4 were injected into the left ventricle of nude mice to establish an intraperitoneal metastasis model. The nude mice were sacrificed in the same way 4 weeks after injection to observe intraperitoneal metastasis, and the differences between the two groups were compared.

### Statistical analysis

The expression level of the DLGAP4 gene in HCC patients was visualised by box plots and scatter plots. The median DLGAP4 expression value was used as a cut-off value for grouping patients based on gene expression. Wilcoxon signed-rank logistic regression was used to analyse the relationship between clinical features of HCC and DLGAP4 expression. The p value was determined using the log-rank test. Univariate and multivariate Cox analyses were used to screen for potential prognostic factors. GraphPad Prism v7.0 software (GraphPad Software, USA) was used to analyse the data. When comparing data between two groups, Student's t test was used to analyse the differences. When more than two groups were compared, one-way analysis of variance (ANOVA) was used to analyse the differences. In all analyses, *, ** and *** indicate P < 0.05, P < 0.01 and P < 0.001, respectively.

### Ethical approval

For human tissues: All methods were carried out in accordance with relevant guidelines and regulations. All experimental protocols were approved by the Ethics Committee of the Second Affiliate Hospital of Nanchang University, China. All of the human tissues used in the present study were obtained with written informed consent from all subjects and their legal guardians. For animals: All experimental protocols were approved by the Animal Ethics Committee of the Second Affiliated Hospital of Nanchang University, China. All methods were carried out in accordance with relevant guidelines and regulations. All methods are reported in accordance with ARRIVE guidelines (https://arriveguidelines.org).

## Results

### High expression of DLGAP4 in HCC

**Figure 1:**
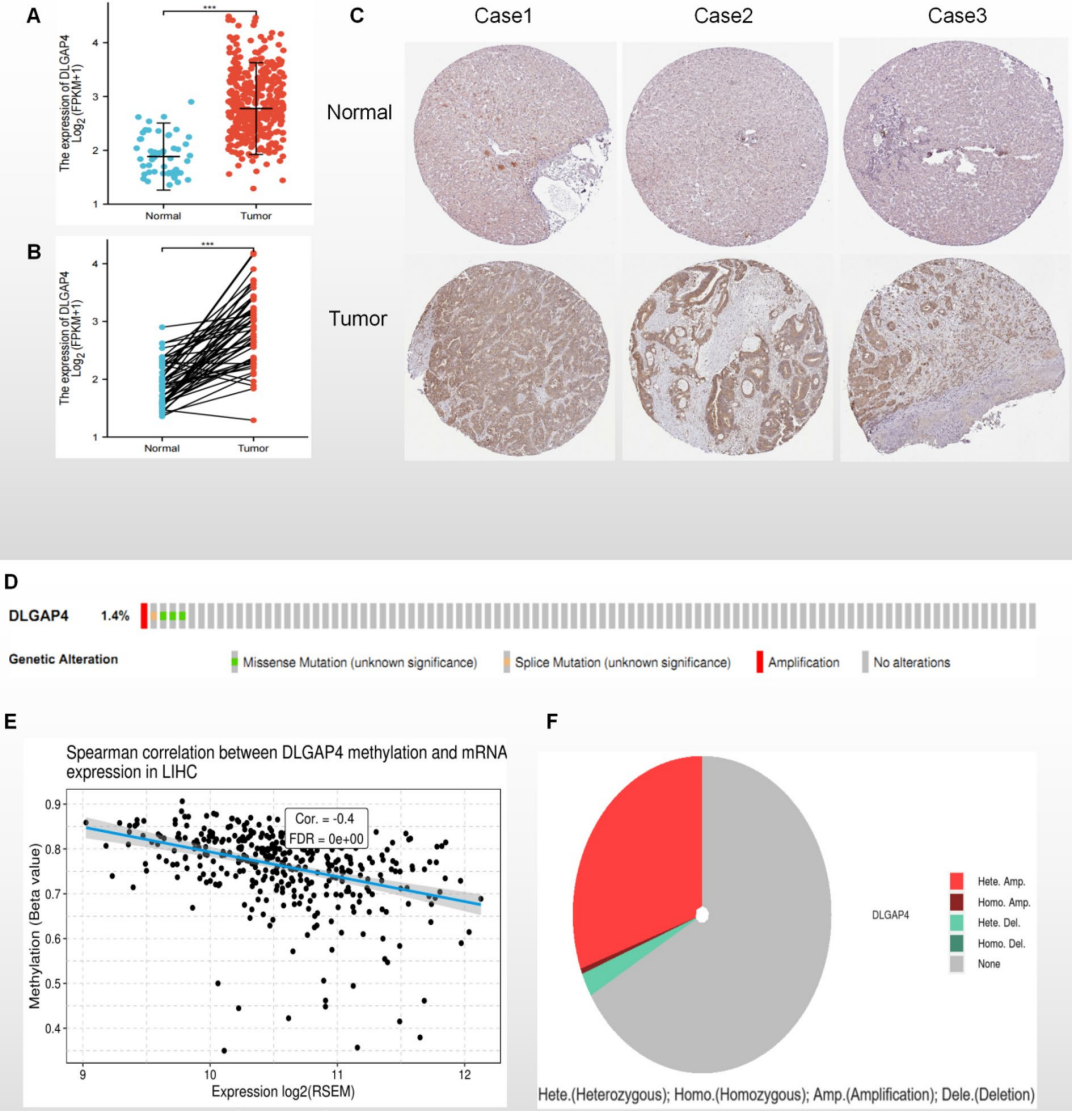
(**A**) DLGAP4 expression in normal and tumour tissues. (**B**) DLGAP4 expression in paired tissues. (**C**) Validation of the expression level of DLGAP4 in HCC using the Human Protein Atlas database (immunohistochemistry). (**D**) The cBioPortal OncoPrint map shows the distribution of DLGAP4 genome changes in HCC patients. (**E**) The GSCA database showed the correlation between methylation and DLGAP4 expression in HCC. (**F**) The GSCA database shows DLGAP4 copy number variation in HCC. *, ** and *** indicate P < 0.05, P < 0.01 and P < 0.001, respectively.

First, using the TCGA database to compare the expression level of DLGAP4 in HCC tissue and normal liver tissue, we found that the expression level of DLGAP4 in HCC tissue was significantly higher than that in normal liver tissue (P < 0.001) (Fig. 1A). This result was consistent in the analysis of paired tissues (P < 0.001) (Fig. 1B). We further verified the expression difference of DLGAP4 between tumour tissues and normal tissues in HCC patients at the protein level based on the HPA database. In the HPA database, DLGAP4 expression was localized in the cytoplasm and cell membrane. DLGAP4 expression was positive in 7 out of 11 HCC specimens. The expression level of DLGAP4 protein in HCC tissues was significantly higher than that in adjacent normal liver tissues (Fig. 1C). Furthermore, we analysed the Oncoprint map of HCC patient genes in the TCGA dataset by using the cBioPortal map (Fig. 1D), and the results showed that gene amplification and missense mutations of DLGAP4 were less than 1.4%. In addition, we analysed the differential expression of DLGAP4 in HCC via the GSCA database and found that it may be related to gene methylation modification and copy number variation (Fig. 1E,F). Overall, DLGAP4 is highly expressed in HCC.

### Correlation of DLGAP4 expression with clinical features

**Figure 2:**
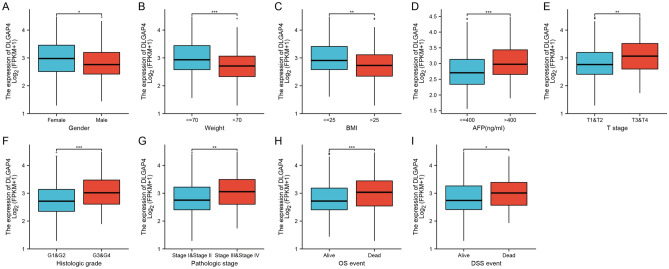
Box plot evaluating DLGAP4 expression in patients with HCC according to different clinical characteristics. (**A**) Gender, (**B**) weight, (**C**) BMI, (**D**) AFP, (**E**) T stage, (**F**) histologic grade, (**G**) pathologic stage, (**H**) OS event, and (**I**) DSS event. *, *** and *** represent P < 0.05, P < 0.01 and P < 0.001, respectively.

We next analysed the relationship between DLGAP4 mRNA expression levels and multiple clinical features of HCC patients based on TCGA database analysis. The results showed that the expression of DLGAP4 was significantly associated with AFP (P = 0.015), tissue grade (P = 0.008), BMI (P = 0.019) and body weight (P = 0.004) (as shown in Table 1). Further analysis showed that the expression of DLGAP4 was significantly associated with sex (P < 0.05), weight (P < 0.001), BMI (P < 0.01), AFP (P < 0.001), T stage (P < 0.01), histological grade (P < 0.001), pathological stage (P < 0.01), OS events (P < 0.001), and DSS events (P < 0.05), as shown in Fig. 2. These findings support that the expression of DLGAP4 is closely related to the clinical characteristics of HCC. However, the expression of DLGAP4 was not significantly associated with other clinical features, including age, height, race, surrounding liver tissue inflammation, vascular invasion, tumour status, residual tumour and progression-free interval (PFI) (see Supplementary Fig. 1A–H).

**Table 1:** Correlation between DLGAP4 expression and the clinicopathological features of the HCC patients.

Characteristic	Low expression of DLGAP4	High expression of DLGAP4	P
n	185	186	
**T stage, n (%)**			0.208
T1	98 (26.6%)	83 (22.6%)	
T2	45 (12.2%)	49 (13.3%)	
T3	39 (9.5%)	45 (12.2%)	
T4	4 (1.1%)	9 (2.4%)	
**N stage, n (%)**			0.126
N0	119 (46.5%)	133 (52%)	
N1	0 (0%)	4 (1.6%)	
**M stage, n (%)**			0.339
M0	122 (45.2%)	144 (53.3%)	
M1	3 (1.1%)	1 (0.4%)	
**Pathologic stage, n (%)**			0.180
Stage I	93 (26.8%)	78 (22.5%)	
Stage II	43 (12.4%)	43 (12.4%)	
Stage III	34 (9.8%)	51 (14.7%)	
Stage IV	3 (0.9%)	2 (0.6%)	
**Tumor status, n (%)**			0.166
Tumor free	108 (30.7%)	93 (26.4%)	
With tumor	69 (19.6%)	82 (23.3%)	
**Gender, n (%)**			0.196
Female	54 (14.6%)	67 (18.1%)	
Male	131 (35.3%)	119 (32.1%)	
**Age, n (%)**			0.751
≤ 60	86 (23.2%)	91 (24.6%)	
> 60	98 (26.5%)	95 (25.7%)	
**Weight, n (%)**			0.004
≤ 70	77 (22.4%)	105 (30.5%)	
> 70	95 (27.6%)	67 (19.5%)	
**BMI, n (%)**			0.019
≤ 25	77 (23%)	100 (29.9%)	
> 25	90 (26.9%)	68 (20.3%)	
**Histologic grade, n (%)**			0.008
G1	37 (10.1%)	18 (4.9%)	
G2	92 (25.1%)	85 (23.2%)	
G3	49 (13.4%)	73 (19.9%)	
G4	5 (1.4%)	7 (1.9%)	
**AFP(ng/ml), n (%)**			0.015
≤ 400	124 (44.6%)	89 (32%)	
> 400	26 (9.4%)	39 (14%)	
Age, median (IQR)	61 (52, 69)	61 (51, 68)	0.707

### High DLGAP4 expression is an independent risk factor for OS

**Figure 3:**
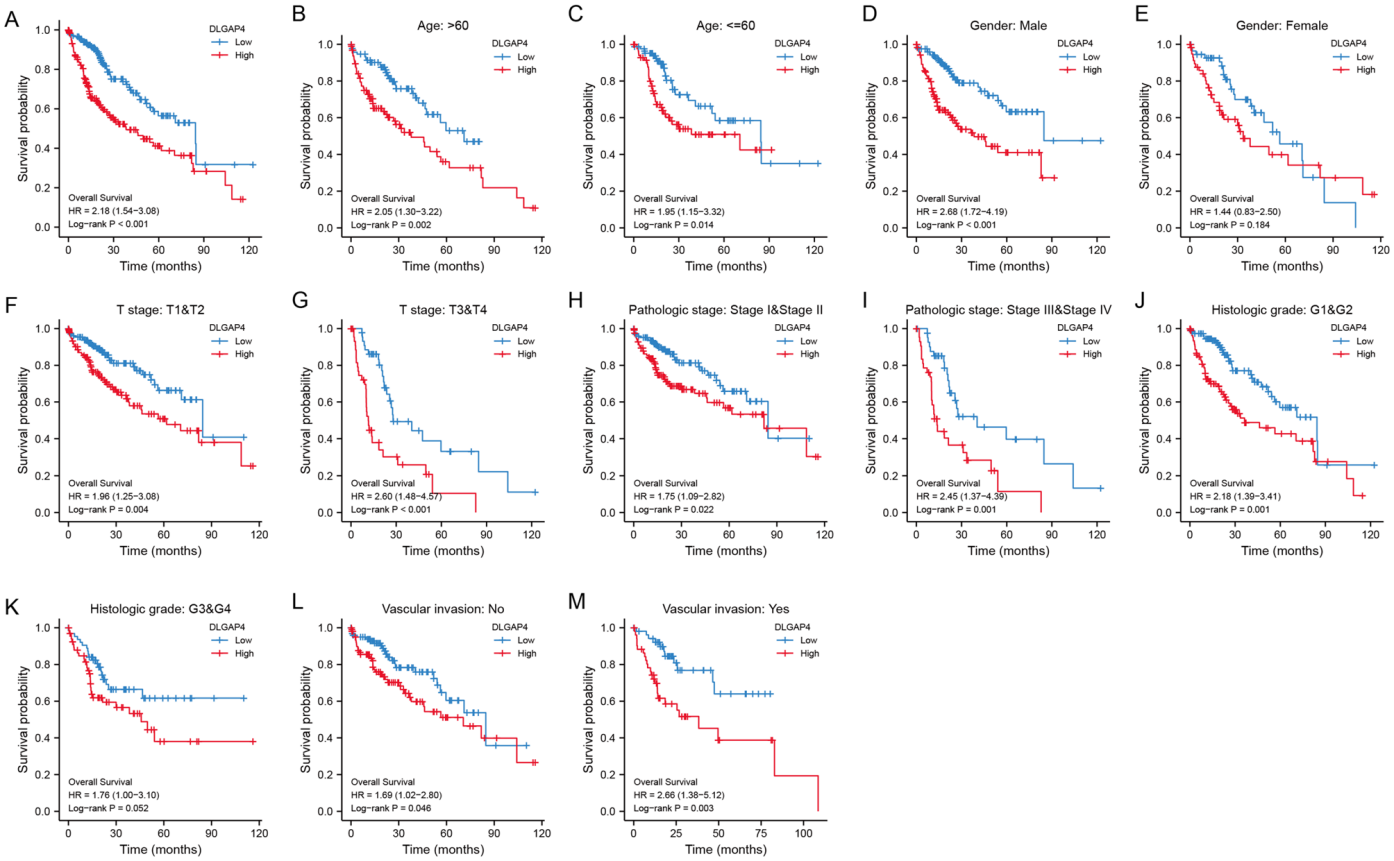
Kaplan‒Meier curve for the OS of HCC patients. (**A**) Kaplan‒Meier curves for all tumour patients grouped by DLGAP4 expression. (**B**–**Q**) Subgroup analysis based on age > 60 years (**B**), age ≤ 60 years (**C**), male (**D**), female (**E**), T1/T2 (**F**), T3/T4 (**G**), stage I/II (**H**), stage III/IV (**I**), G1/G2 (**J**), G3/G4 (**K**), vascular invasion: no (**L**), vascular invasion: yes (**M**). P < 0.05 indicates statistical significance.

Kaplan‒Meier survival analysis showed that high DLGAP4 expression was associated with poor prognosis (P < 0.001), as shown in Fig. 3A. Subgroup analysis based on different clinical characteristics showed that age > 60 years (P = 0.002), age ≤ 60 years (P = 0.014), male sex (P < 0.001), female sex (P = 0.184), T1/T2 (P = 0.004), T3/T4 (P < 0.001), stage I/II (P = 0.022), stage III/IV (P = 0.001), G1/G2 (P = 0.001), G3/G4 (P = 0.052), vascular invasion: no (P = 0.046), vascular invasion: yes (P = 0.003), and high DLGAP4 expression were significantly associated with a poor prognosis, as shown in Fig. 3B–M. Univariate Cox analysis showed that high DLGAP4 expression was significantly associated with poor OS (hazard ratio [HR] = 2.193, 95% CI 1.529–3.146, P < 0.001). Multivariate Cox analysis confirmed that DLGAP4 gene expression was an independent risk factor for OS in HCC patients (HR = 2.273, 95% CI 1.546–3.341, P < 0.001) (as shown in Table 2).

**Table 2:** Univariate and multivariate Cox regression analyses of the clinical characteristics associated with OS in HCC.

Characteristics	Total (N)	Univariate analysis		Multivariate analysis	
		HR (95% CI)	P value	HR (95% CI)	P value
**Age**	373				
≤ 60	177	Reference			
> 60	196	1.205 (0.850–1.708)	0.295		
**Gender**	373				
Female	121	Reference			
Male	252	0.793 (0.557–1.130)	0.200		
**T stage**	370				
T1 & T2	277	Reference			
T3 & T4	93	2.598 (1.826–3.697)	< 0.001	2.406 (0.329–17.600)	0.387
**Pathologic stage**	349				
Stage I & Stage II	259	Reference			
Stage III & Stage IV	90	2.504 (1.727–3.631)	< 0.001	1.060 (0.146–7.697)	0.954
**Histologic grade**	368				
G1 & G2	233	Reference			
G3 & G4	135	1.091 (0.761–1.564)	0.636		
**AFP (ng/ml)**	279				
≤ 400	215	Reference			
> 400	64	1.075 (0.658–1.759)	0.772		
**Vascular invasion**	317				
No	208	Reference			
Yes	109	1.344 (0.887–2.035)	0.163		
**DLGAP4**	373				
Low	186	Reference			
High	187	2.193 (1.529–3.146)	< 0.001	2.273 (1.546–3.341)	< 0.001

### Diagnostic value of DLGAP4 expression in HCC

**Figure 4:**
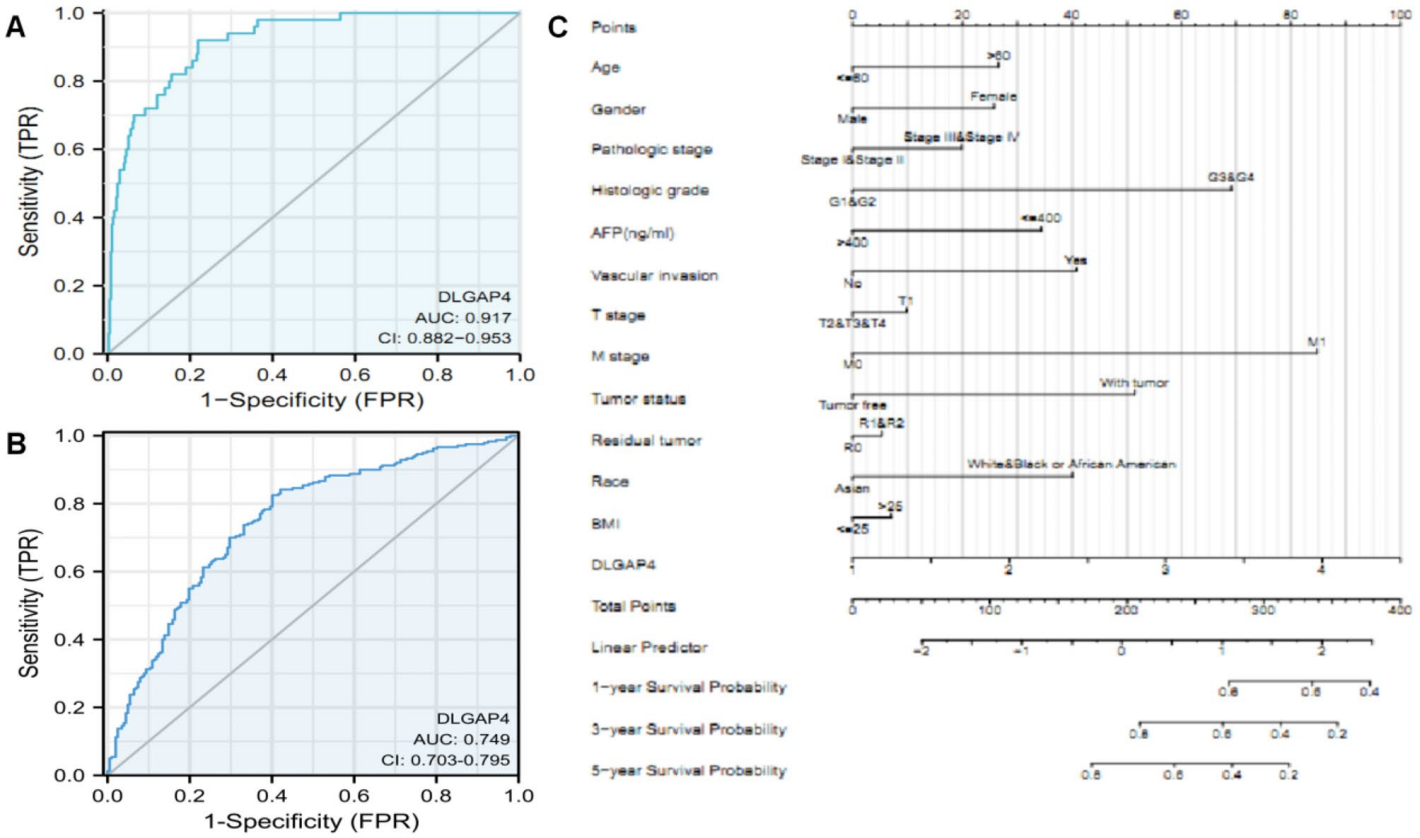
Diagnostic value of DLGAP4 expression in HCC. (**A**, **B**) ROC curve analysis for DLGAP4 expression in HCC and adjacent tissue used by TCGA and ICGC. (**C**) Nomogram survival prediction chart for predicting the 1-, 3-, and 5-year OS rates.

It has been reported in the literature that ROC curves and nomograms can be used to predict the value of gene expression in cancer diagnosis and survival probability^24^. To this end, we used ROC curve analysis combined with nomogram analysis to evaluate the diagnostic value of DLGAP4 expression in HCC patients and predict the survival probability of patients at 1, 3, and 5 years. ROC analysis based on the TCGA database showed that the area under the ROC curve (AUC) was 0.917, indicating high diagnostic value (Fig. 4A). Similarly, the ICGC database analysis showed that the AUC was 0.749, indicating high diagnostic value (Fig. 4B). Additionally, we constructed a nomogram by combining clinical variables with DLGAP4 to predict the 1-, 3-, and 5-year survival probabilities of patients. The results showed that the prediction of prognosis based on DLGAP4 expression level was better than that based on traditional methods, such as AFP, pathological stage, T stage, M stage, tumour status, residual tumour, age, and sex (Fig. 4C).

**Figure 5:**
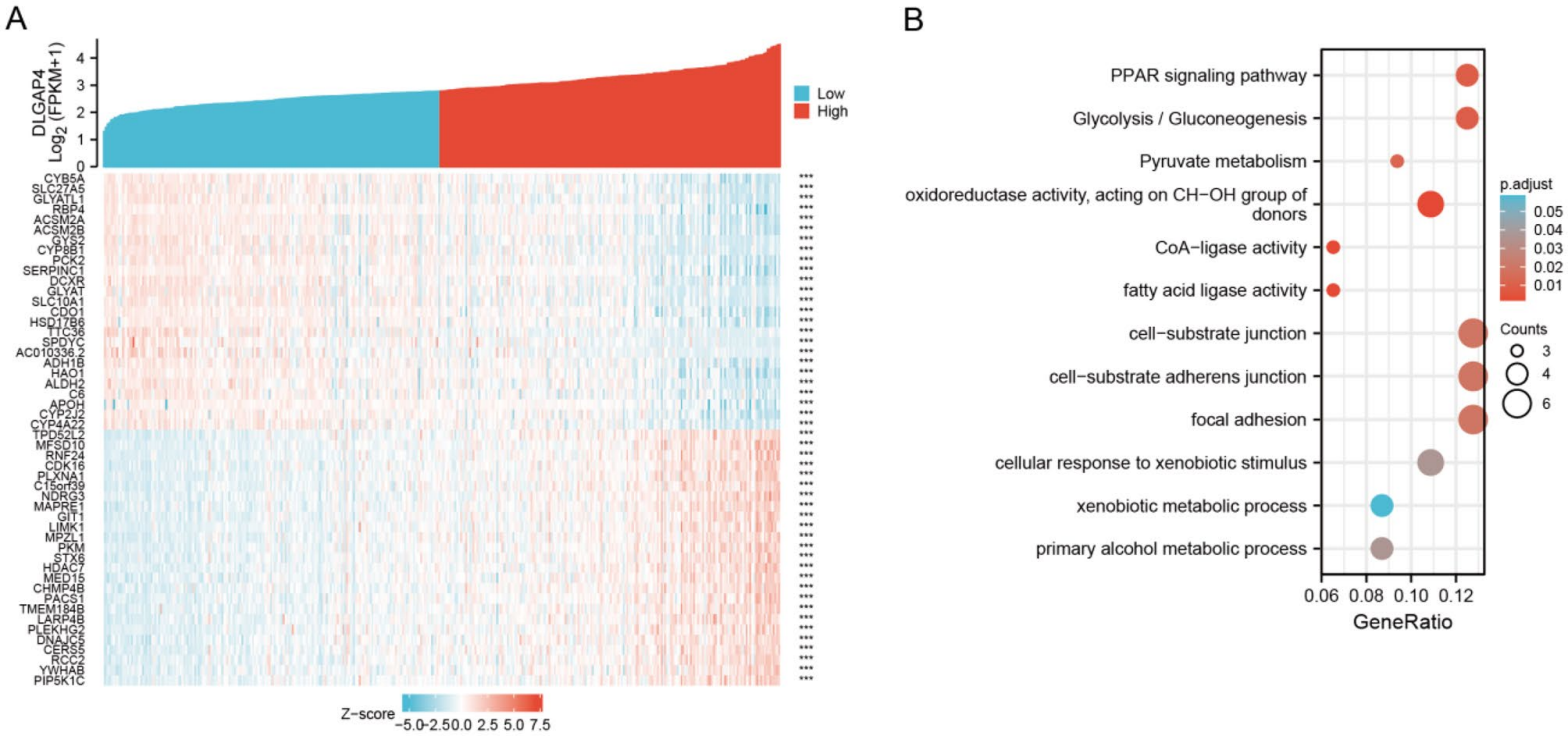
DLGAP4 functional clustering and interaction network analysis of DLGAP4-related genes. (**A**) Heatmap showing the top 50 genes in HCC that were positively and negatively related to DLGAP4. Red represents positively related genes, and blue represents negatively related genes. (**B**) Gene Ontology (GO) term and Kyoto Encyclopedia of Genes and Genomes (KEGG) pathway analyses of DLGAP4-related genes in HCC.

By examining the TCGA database using the LinkFinder module of the LinkedOmics website, we detected the genes coexpressed with DLGAP4 in HCC and plotted them as a heatmap to further understand the biological function of DLGAP4 in HCC. Green dots indicate the bottom 25 genes negatively correlated with DLGAP4, and red dots indicate the top 25 genes positively correlated with DLGAP4 (Fig. 5A). Next, we used DAVID functional annotation and bioinformatics microarray analysis to determine the enriched GO terms and KEGG pathways among the 50 genes related to DLGAP4 mentioned above and found that these genes were significantly enriched in the PPAR signalling pathway, glycolysis/gluconeogenesis and pyruvate metabolism (Fig. 5B).

### DLGAP4 is abnormally highly expressed in clinical tissues and cell lines

**Figure 6:**
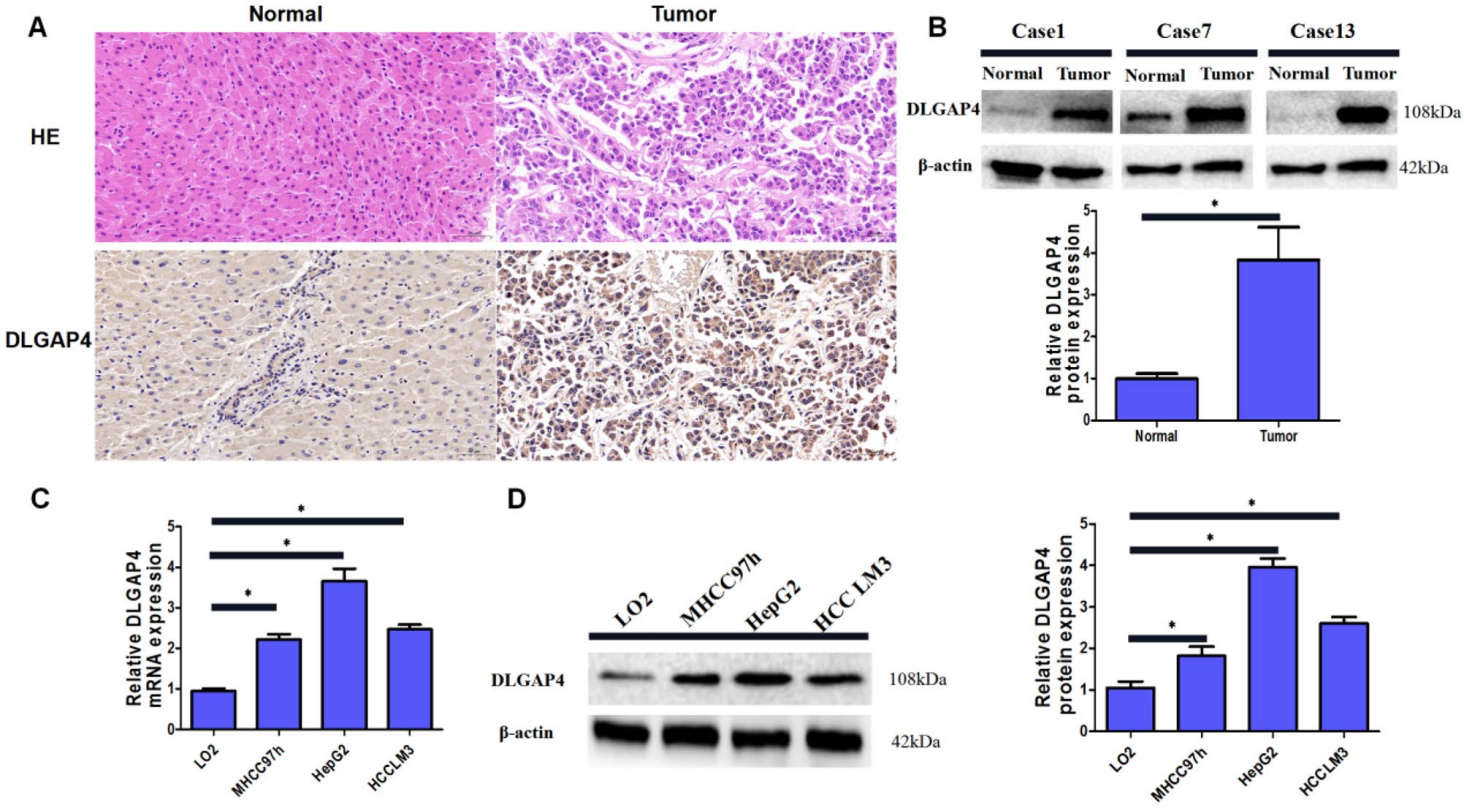
The expression of DLGAP4 in clinical specimens and cell lines of HCC. (**A**) Representative images of DLGAP4 staining in HCC tissues and adjacent normal tissues shown by haematoxylin–eosin staining (HE) and immunohistochemistry (IHC). (**B**) Western blot indicating the expression of DLGAP4 in HCC tissues and matching adjacent normal tissues. β-actin was used as an internal control. (**C**, **D**) mRNA and protein levels of DLGAP4 in a normal live cell line and two human HCC cell lines detected by RT–qPCR and Western blot, respectively. The data were obtained from the average of three independent experiments. *P < 0.05.

To confirm our bioinformatics results, we used immunohistochemistry (IHC) to verify the differential expression of DLGAP4 in HCC tissues and normal liver tissues. We assessed 20 pairs of HCC tissues and their corresponding adjacent normal liver tissues. IHC staining showed that DLGAP4 was mainly expressed in the cytoplasm and cell membrane. Compared with that in normal liver tissue, the expression of DLGAP4 in HCC was abnormally elevated (Fig. 6A). We used Western blotting to detect the expression of DLGAP4 in 20 pairs of HCC and paired adjacent normal liver tissues. The results showed that the expression of DLGAP4 protein in HCC tissues was significantly higher than that in normal liver tissues (Fig. 6B). In addition, we used qRT‒PCR and Western blotting to detect the expression of DLGAP4 mRNA and protein in HCC cell lines (MHCC97h, HCCLM3, HepG2) and normal liver cells (LO2), and the results showed that the expression of DLGAP4 in HCC cell lines was significantly higher than that in normal liver cells (P < 0.05) (Fig. 6C,D). Moreover, we found that the results of both qRT‒PCR and Western blotting indicated that DLGAP4 expression was relatively high in HepG2 and HCCLM3 cells. Therefore, HepG2 and HCCLM3 cells were selected to construct stable DLGAP4 knockdown cell lines, and the DLGAP4 overexpression plasmid was transfected into MHCC97h cells for subsequent functional experiments.

### DLGAP4 promotes the proliferation and migration of HCC cells

**Figure 7:**
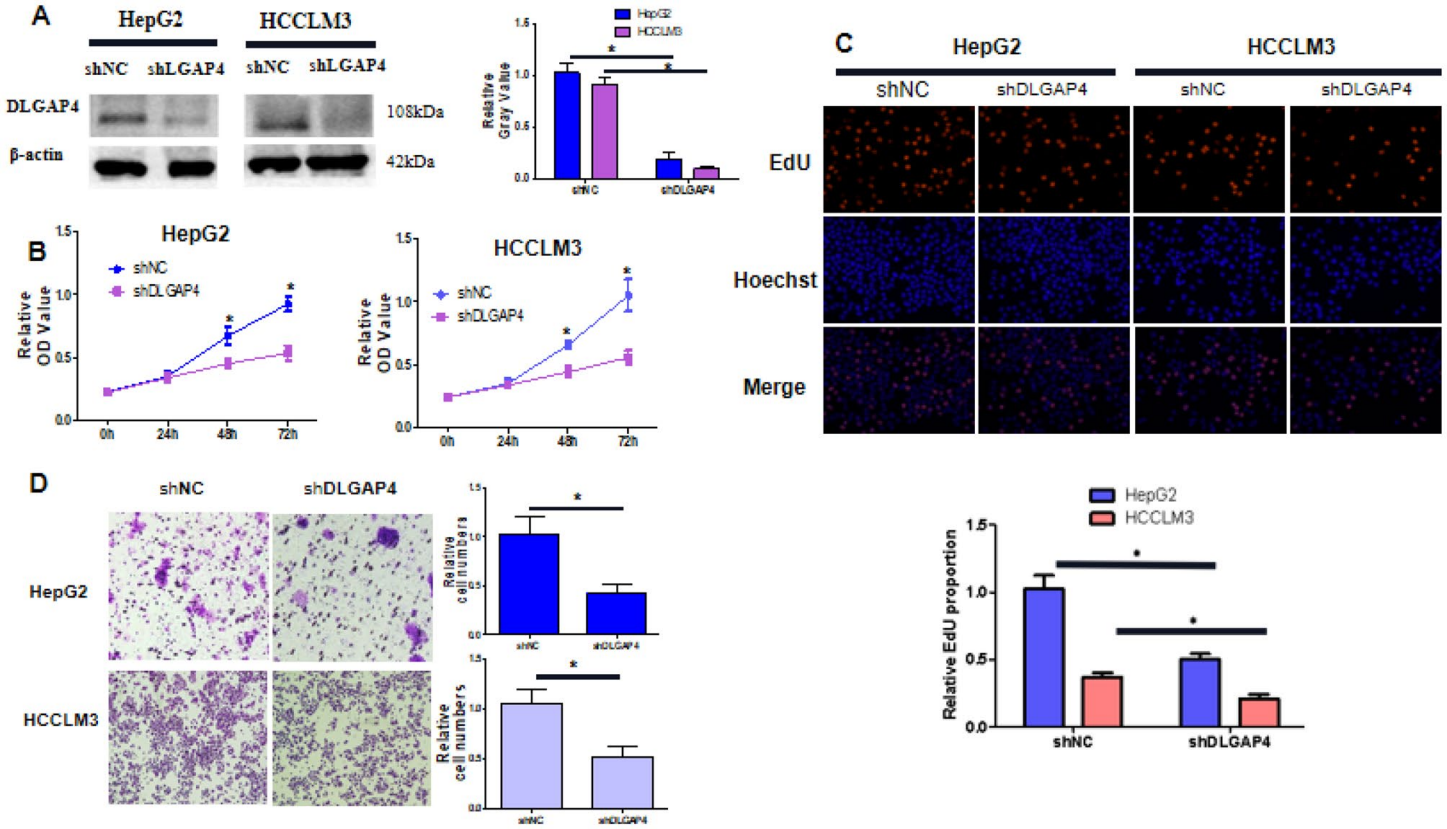
DLGAP4 knockdown suppresses the proliferation and growth of HCC cells in vitro. (**A**) Western blot analyses showing the expression levels of DLGAP4 in HepG2 and HCCLM3 cells stably transfected with shDLGAP4 or shNC. β-actin was used as a loading control. (**B**) CCK-8 assays revealed that shDLGAP4 inhibited the proliferation of HepG2 and HCCLM3 cells. (**C**) Representative micrographs of BrdU incorporation in shDLGAP4 and control cells. (**D**) Transwell migration assays of HepG2 cells and HCCLM3 cells transfected with shNC or shDLGAP4. The data were obtained from the average of three independent experiments. *P < 0.05.

First, to verify the effect of DLGAP4 on the biological behaviour (proliferation, migration and metastasis) of HCC cells, we constructed HepG2 and HCCLM3 cell lines with stable DLGAP4 knockdown and verified the transfection efficiency by Western blotting. The results showed that after transfection of shDLGAP4, the expression of DLGAP4 in HepG2 and HCCLM3 cell lines was significantly decreased (P < 0.05) (Fig. 7A). Next, in this study, the effect of shDLGAP4 on the proliferation ability of HCC cells was detected by CCK-8 and EdU experiments. The results showed that after interfering with the expression of DLGAP4 in HepG2 and HCCLM3 cells, proliferation was significantly inhibited (P < 0.05) (Fig. 7B,C). Second, we used a Transwell assay to evaluate the effect of DLGAP4 knockdown on the migration of HCC cells. The results showed that the downregulation of DLGAP4 significantly inhibited the migration ability of HepG2 cells and HCCLM3 cells (P < 0.05) (Fig. 7D). In addition, to further verify the effect of DLGAP4 overexpression on the biological behaviour of HCC cells, we constructed the DLGAP4 overexpression plasmid DLGAP4-flag and the overexpression control plasmid Vector-flag. The HCC cell line MHCC97 was transfected with DLGAP4-flag or Vector-flag, and the transfection effect was detected by Western blotting (Supplementary Fig. 2A). CCK-8 and EdU experiments showed that overexpression of DLGAP4 promoted the proliferation of MHCC97h cells (Supplementary Fig. 2B,C). Transwell assays showed that overexpression of DLGAP4 promoted the invasion and metastasis of MHCC97h cells (Supplementary Fig. 2D). Therefore, DLGAP4 promoted the proliferation, invasion and metastasis of HCC cells.

### In vivo experiments

**Figure 8:**
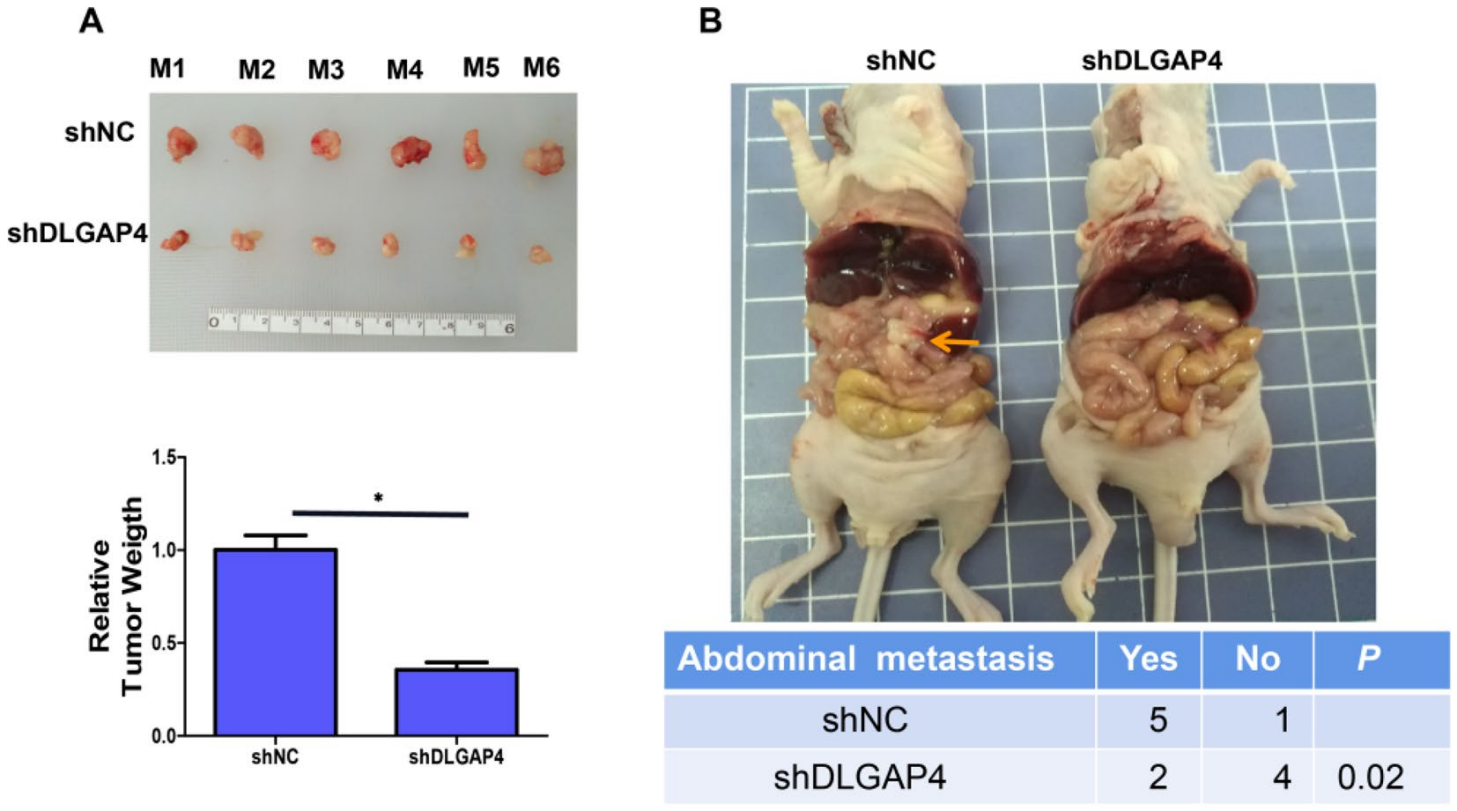
DLGAP4 knockdown suppresses the proliferation and growth of HCC cells in vivo. (**A**) Cells transfected with shNC or shDLGAP4 were injected into mice. Mice were sacrificed after 4 weeks. Tumours were harvested from sacrificed mice, and their weights were measured and compared between the two groups. (**B**) HepG2 cells transfected with shDLGAP4 or shNC were injected into the left ventricle of nude mice. Mice were sacrificed after 4 weeks. Representative images of abdominal metastases. Abdominal metastases and those without metastases are shown in this table. The data were obtained from the average of three independent experiments. *P < 0.05.

Through animal experiments, we further evaluated the effect of DLGAP4 on HCC proliferation and metastasis. In this study, HCC xenograft tumour models were successfully constructed, and a representative image of the tumour is shown in Fig. 8A. The results showed that the tumour weight formed by shDLGAP4 in HepG2 cells was significantly smaller than that formed by shNC cells. The intraperitoneal metastasis model of HCC was constructed by injection into the left ventricle and compared with the intraperitoneal metastasis of the tumour. The results showed that the intraperitoneal metastasis of the shDLGAP4 group was significantly reduced (Fig. 8B). The above results indicated that downregulation of DLGAP4 inhibited tumour proliferation, migration and metastasis.

### DLGAP4 regulates the PPAR signalling pathway

**Figure 9:**
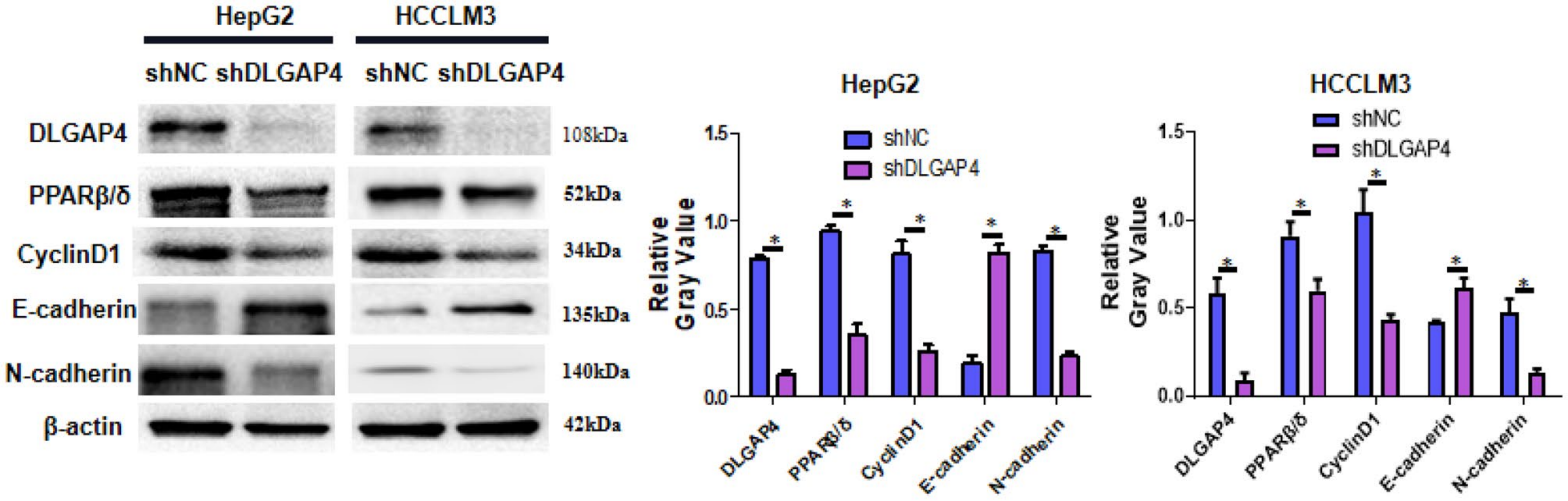
Interfering with DLGAP4 inhibits the PPARβ/δ signalling pathway and the expression of proliferation- and metastasis-related proteins. Western blotting was performed to measure the protein expression of DLGAP4, PPARβ/δ, CyclinD1, E-cadherin and N-cadherin in shNC or shDLGAP4 HepG2 and HCCLM3 cells. The data were obtained from the average of three independent experiments. *P < 0.05.

Bioinformatics analysis has shown that high expression of DLGAP4 is accompanied by enrichment of the PPAR signalling pathway in HCC. It has been reported in the literature that the PPAR signalling pathway is closely related to the proliferation, invasion and metastasis of cancer^25,26^. As a subtype of PPARs, PPARβ/δ is closely related to the proliferation and metastasis of cancer. Cyclin D1 is involved in the regulation of cell proliferation, the cell cycle and other life activities and plays an important role in affecting the normal physiological functions of cells^27^. An increasing number of studies have shown that cyclin D1 plays an important role in tumour proliferation^28,29^. E-cadherin and N-cadherin regulate intercellular adhesion and are important markers of tumour metastasis^30^. To verify whether DLGAP4 regulates the PPARβ/δ pathway, we measured the expression of PPARβ/δ and proliferation- and metastasis-related proteins in the shDLGAP4 group and shNC group by Western blotting. The results showed that compared with the shNC groups, the expression of PPARβ/δ, N-cadherin and cyclin D1 in the ShDLGAP4 group was significantly decreased, and the expression of E-cadherin was significantly increased (P < 0.05) (Fig. 9). However, after transfection of DLGAP4-flag into the HCC cell line MHCC97h, the expression levels of PPARβ/δ, N-cadherin and cyclin D1 were significantly increased, while the expression of E-cadherin was significantly decreased compared with that in the vector-flag group (Supplementary Fig. 3). In conclusion, DLGAP4 regulates proliferation- and metastasis-related proteins in HCC cells by regulating the PPARβ/δ signalling pathway.

## Discussion

The DLGAP protein family includes a total of 5 members. At present, cancer research mainly focuses on DLGAP5. DLGAP5 overexpression promotes tumour proliferation and metastasis in HCC^31^, endometrial cancer^32^, pancreatic cancer^33^, colorectal cancer^34^, ovarian cancer^35^, and breast cancer^36^. DLGAPs are highly conserved in molecular structure and function. Liu^37^ and others believed that DLGAP4 may have similar cancer-promoting functions as DLGAP5. They found that the expression of DLGAP4 in gastric cancer tissue was significantly higher than that in normal gastric tissue. There is a clear correlation between high expression of DLGAP4 and a poor prognosis for gastric cancer patients, indicating that it can be used as a potential prognostic marker. However, to date, there have been no studies on the clinical significance of DLGAP4 in HCC. This study found that the expression of DLGAP4 in HCC was significantly higher than that in the corresponding adjacent normal liver tissue. This result was further confirmed by qRT‒PCR, Western blotting and immunohistochemistry. Moreover, the expression of DLGAP4 is closely related to the clinicopathological characteristics of HCC patients. This study shows that high DLGAP4 expression is an independent risk factor for poor prognosis in HCC patients. Therefore, DLGAP4 may play a tumour-promoting role in HCC patients.

The aberrant expression of genes has value in diagnosing cancer and predicting survival^24^. Jia Hui et al. evaluated the importance of GPI in the diagnosis and survival rate prediction of lung adenocarcinoma through ROC curve analysis combined with nomogram analysis^21^. In this study, this approach indicated that high DLGAP4 expression has a high value in the diagnosis of HCC patients and in predicting the 1-year, 3-year and 5-year survival probability of patients. Clinicopathological features are important indicators of survival and prognosis in patients with HCC. AFP is a classic indicator for predicting the survival and prognosis of patients with HCC^38^. Interestingly, we found that DLGAP4 performed better than clinicopathological features such as AFP in predicting the 1-, 3-, and 5-year survival rates of HCC patients.

Studies have revealed that mutations in the CPG island of DLGAP4 located in the promoter region may lead to epigenetic changes^10^. Through cBioPortal analysis^39,40^, we found that DLGAP4 had related mutations in HCC that may be located in CPG islands in the promoter region. This mutation may increase the expression of transcript variants of DLGAP4, resulting in abnormally high expression of DLGAP4, which in turn leads to the development of HCC. In addition, ICGC database analysis showed that gene copy number variation and methylation may also be involved in the differential expression of DLGAP4 in HCC.

As a scaffold protein, DLGAP4 plays a key role in regulating synaptic development, function and plasticity^6^. Studies have shown that promoter methylation leads to abnormal expression of DLGAP4, which leads to cerebellar ataxia^10^. However, the role of DLGAP4 in cancer has rarely been studied. Although Liu's^37^ bioinformatics analysis showed that DLGAP4 may play an oncogenic role in gastric cancer and may become a prognostic indicator for predicting gastric cancer, it has not been verified in in vivo and in vitro functional experiments. On the basis of bioinformatics analysis, we found through in vitro and in vivo functional experiments that interfering with DLGAP4 expression inhibited the proliferation, migration and metastasis of HCC cells. Therefore, DLGAP4 can promote the progression of HCC and may play a biological function role in HCC progression.

The PPAR signalling pathway is involved in the regulation of cell proliferation, invasion, and metastasis and is a popular pathway in tumour research^41^. PPARβ/δ is a major subtype of PPARs. Studies have found that PPARβ/δ is highly expressed in tumours and can promote the proliferation of tumour cells. PPARβ/δ is abnormally highly expressed in epithelial ovarian cancer^42^, colon cancer^26^, and liposarcoma^43^. Other studies have found that PPARβ/δ is closely related to the invasion and metastasis of cancer. Ham et al.^25^ found that PPARβ/δ can upregulate Snail expression and downregulate E-cadherin to promote the metastasis of invasive melanoma. Yoshinaga^44^ found that colorectal cancer patients with high expression of PPARβ/δ had an increased risk of liver metastasis, leading to poor prognosis. In addition, the PPARβ/δ signalling pathway plays a key role in mediating the progression of HCC, and activation of the PPARβ/δ signalling pathway promotes the proliferation^16^, invasion and metastasis of human HCC cells^45^. In this study, we found that the high expression of DLGAP4 was accompanied by the enrichment of the PPAR signalling pathway in HCC. We further confirmed that interfering with DLGAP4 expression can inhibit the expression of PPARβ/δ, upregulate E-cadherin, and downregulate CyclinD1 and N-cadherin. Therefore, DLGAP4 promotes HCC cell proliferation, invasion and metastasis through the regulation of the PPARβ/δ signalling pathway.

In conclusion, we found that DLGAP4 is upregulated in HCC and that high DLGAP4 expression is associated with the clinical progression of HCC and is an independent risk factor for OS in HCC patients. We also found that DLGAP4 promotes the proliferation and invasion of HCC cells and has potential application value in the diagnosis and prognosis evaluation of HCC. In addition, the PPARβ/δ signalling pathway may mediate the cancer-promoting effect of DLGAP4 in HCC.

Despite our systematic analysis of DLGAP4 and cross-validation using different databases, there are still shortcomings in this study. On the one hand, the data collected in the database may be imperfect, and there may be data collection bias. On the other hand, we do not know the specific mechanism by which DLGAP4 regulates the PPARβ/δ signalling pathway in HCC, which needs to be further clarified in further studies.

## Supplementary Information

Supplementary Figure 1.

Supplementary Figure 2.

Supplementary Figure 3.

Supplementary Information 4.

Supplementary Information 5.

Supplementary Information 6.

Supplementary Information 7.

## Data Availability

All data generated or analysed during this study are included in this article. We have not used other data that have already been published. All the data presented in this article are original results derived from this study.

## Abbreviations

DLGAP4: Disc large associated protein 4
HCC: Hepatocellular carcinoma
TCGA: The Cancer Genome Atlas
ICGC: International Cancer Genome Consortium
GSCA: Gene Set Cancer Analysis
OS: Overall Survival
DSS: Disease Free Survival
PFI: Progression Free Interval
PPAR: Peroxisome proliferator-activated receptor
HR: Hazard ratio
ROC: Receiver Operating Characteristic
AUC: Area under the curve
KEGG: Kyoto Encyclopedia of Genes and Genomes
GO: Gene Ontology
