# Supplementary material for: DLGAP4 acts as an effective prognostic predictor for hepatocellular carcinoma and is closely related to tumour progression
Source: Sci Rep. 2022 Nov 17;12:19775. doi: 10.1038/s41598-022-23837-y (PMC9672105; doi:10.1038/s41598-022-23837-y)
Supplement: Supplementary file 1 — Supplementary Figure 1. [file 41598_2022_23837_MOESM1_ESM.docx]

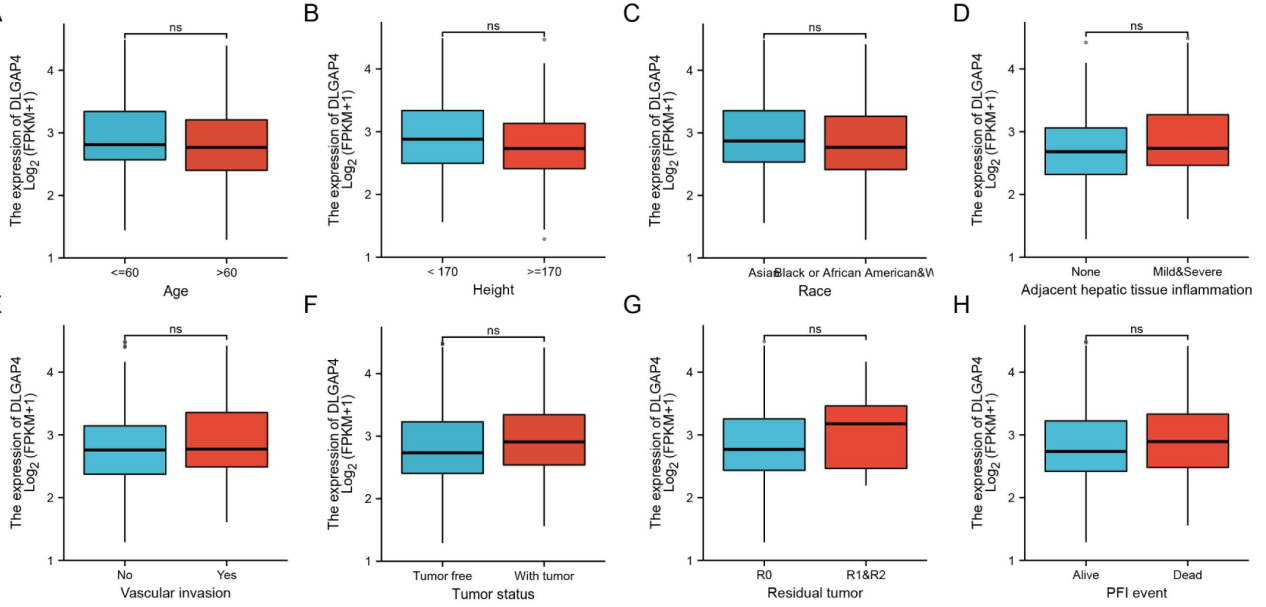


Supplementary Figure 1. Box plot showing DLGAP4 expression in patients with HCC according to different clinical characteristics. (A) Age, (B) height, (C) race, (D) adjacent hepatic tissue inflammation, (E) vascular invasion, (F) tumour status, (G) residual tumour, and (H) PFI event. NS indicates no significant difference.
