# Supplementary material for: DLGAP4 acts as an effective prognostic predictor for hepatocellular carcinoma and is closely related to tumour progression
Source: Sci Rep. 2022 Nov 17;12:19775. doi: 10.1038/s41598-022-23837-y (PMC9672105; doi:10.1038/s41598-022-23837-y)
Supplement: Supplementary file 2 — Supplementary Figure 2. [file 41598_2022_23837_MOESM2_ESM.docx]

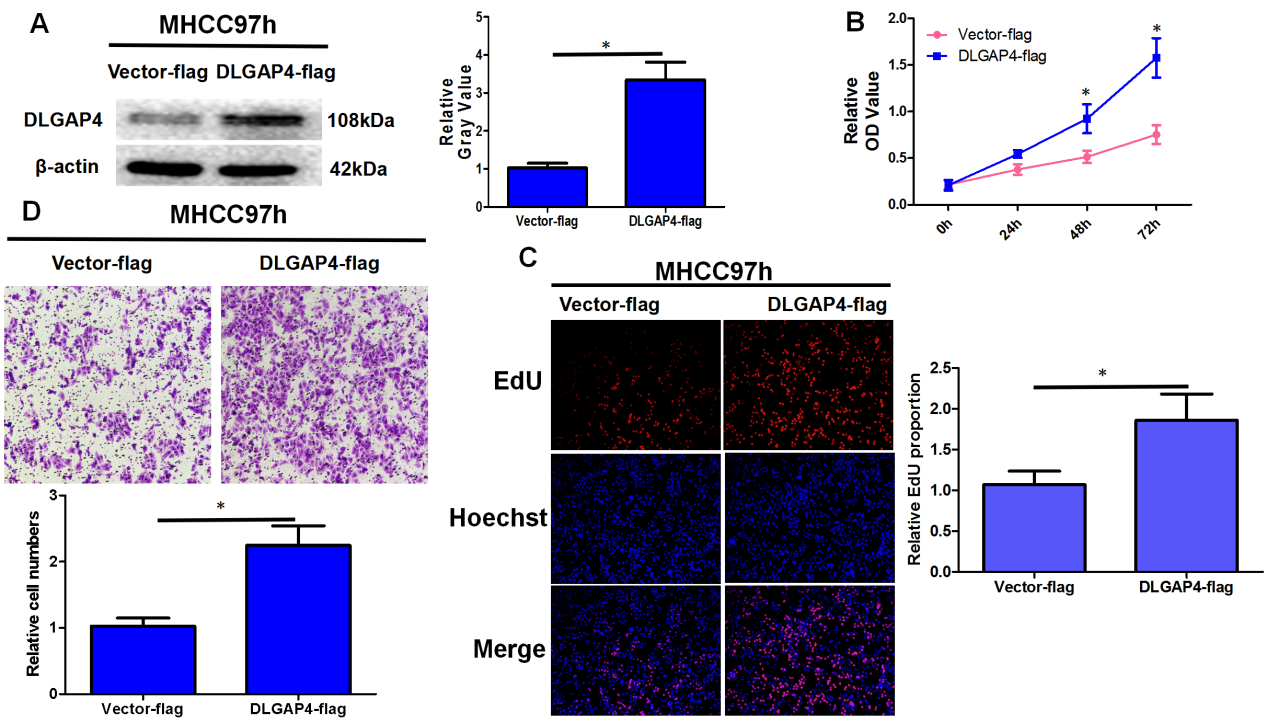


Supplementary Figure 2. Overexpression of DLGAP4 promotes the proliferation and migration of HCC cells in vitro. (A) Western blot showing the expression level of DLGAP4 in MHCC97h cells stably transfected with vector-flag and DLGAP4-flag. β-actin was used as an internal control. (B) CCK-8 assays revealed that DLGAP4 promoted proliferation of MHCC97h cells. (C) EdU was used to detect the proliferation activity of MHCC97h cells. (D) Transwell migration assays of MHCC97h cells transfected with vector-flag or DLGAP4-flag. The data represent the average of three independent experiments. * represents P<0.05.
