# Supplementary material for: DLGAP4 acts as an effective prognostic predictor for hepatocellular carcinoma and is closely related to tumour progression
Source: Sci Rep. 2022 Nov 17;12:19775. doi: 10.1038/s41598-022-23837-y (PMC9672105; doi:10.1038/s41598-022-23837-y)
Supplement: Supplementary file 3 — Supplementary Figure 3. [file 41598_2022_23837_MOESM3_ESM.docx]

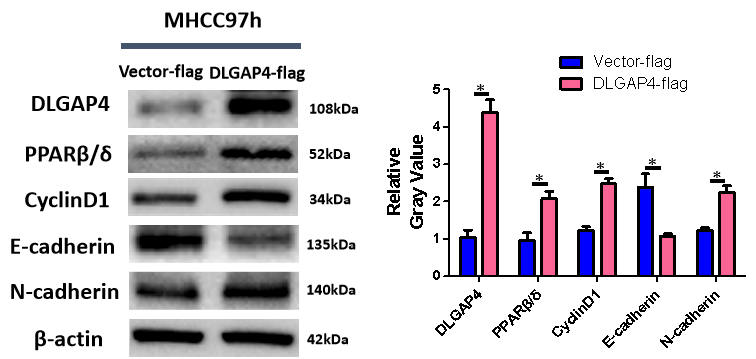


Supplementary Figure 3. Overexpression of DLGAP4 promoted the expression of PPARβ/δ signalling pathway- and proliferation- and metastasis-related proteins. Western blotting was performed to measure the protein expression of DLGAP4, PPARβ/δ, cyclin D1, E-cadherin and N-cadherin in vector-flag or DLGAP4-flag MHCC97h cells. The data represent the average of three independent experiments. * represents P<0.05.
