# Supplementary figures and images for: DLGAP4 acts as an effective prognostic predictor for hepatocellular carcinoma and is closely related to tumour progression
Source: Sci Rep. 2022 Nov 17;12:19775. doi: 10.1038/s41598-022-23837-y (PMC9672105; doi:10.1038/s41598-022-23837-y)

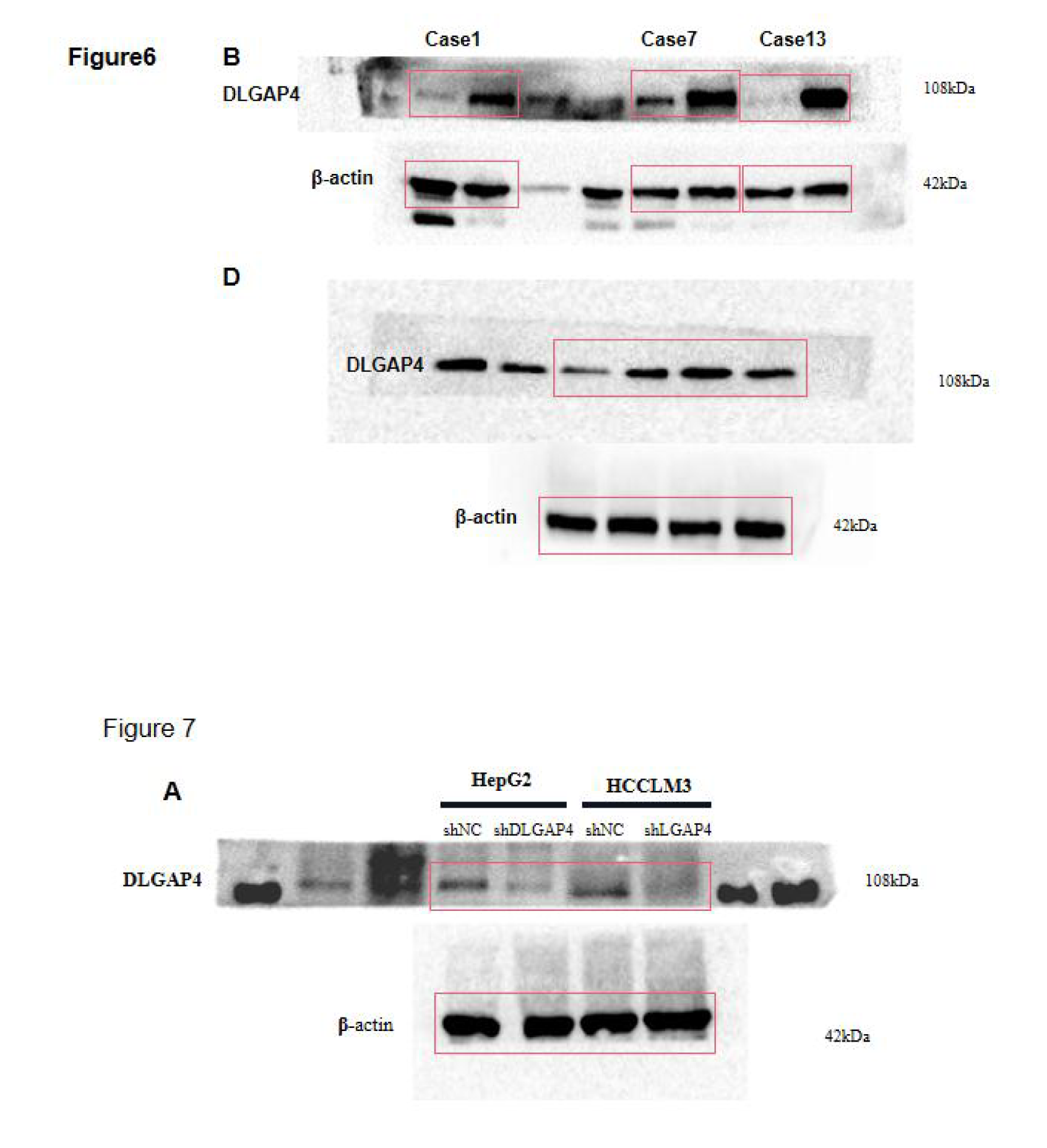

Supplement: Supplementary file 4 — Supplementary Information 4. [file 41598_2022_23837_MOESM4_ESM.tif]

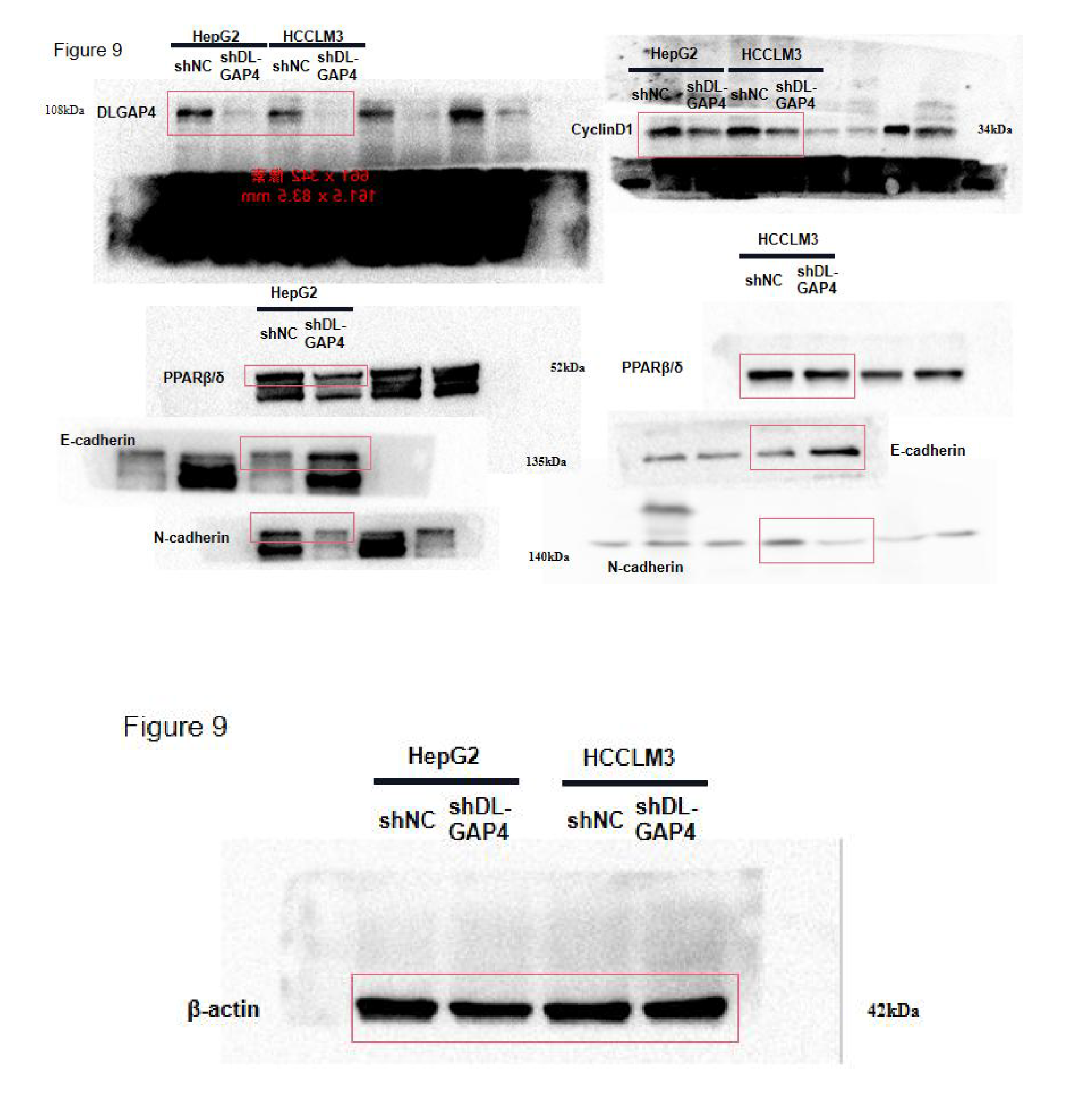

Supplement: Supplementary file 5 — Supplementary Information 5. [file 41598_2022_23837_MOESM5_ESM.tif]

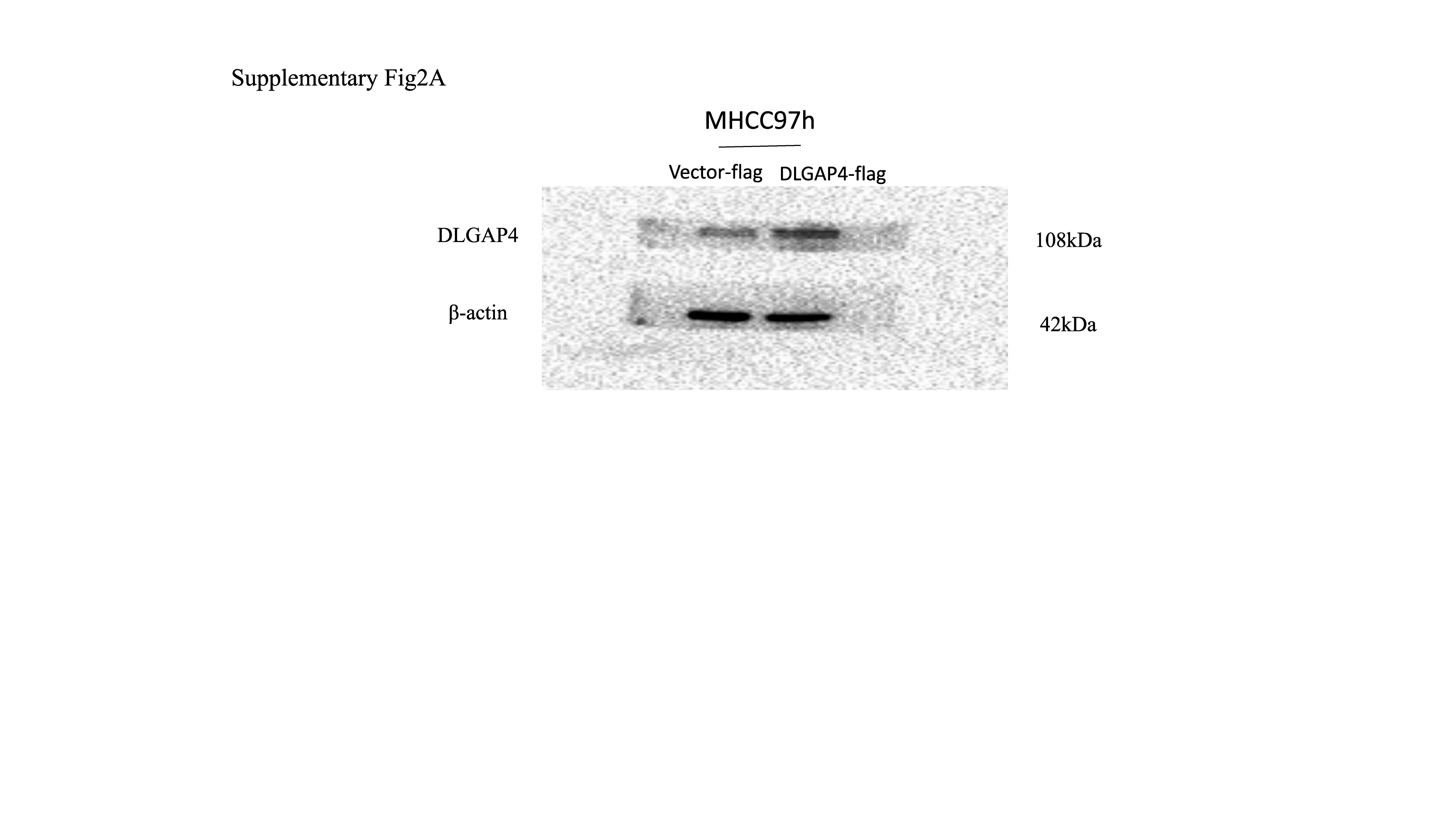

Supplement: Supplementary file 6 — Supplementary Information 6. [file 41598_2022_23837_MOESM6_ESM.tif]

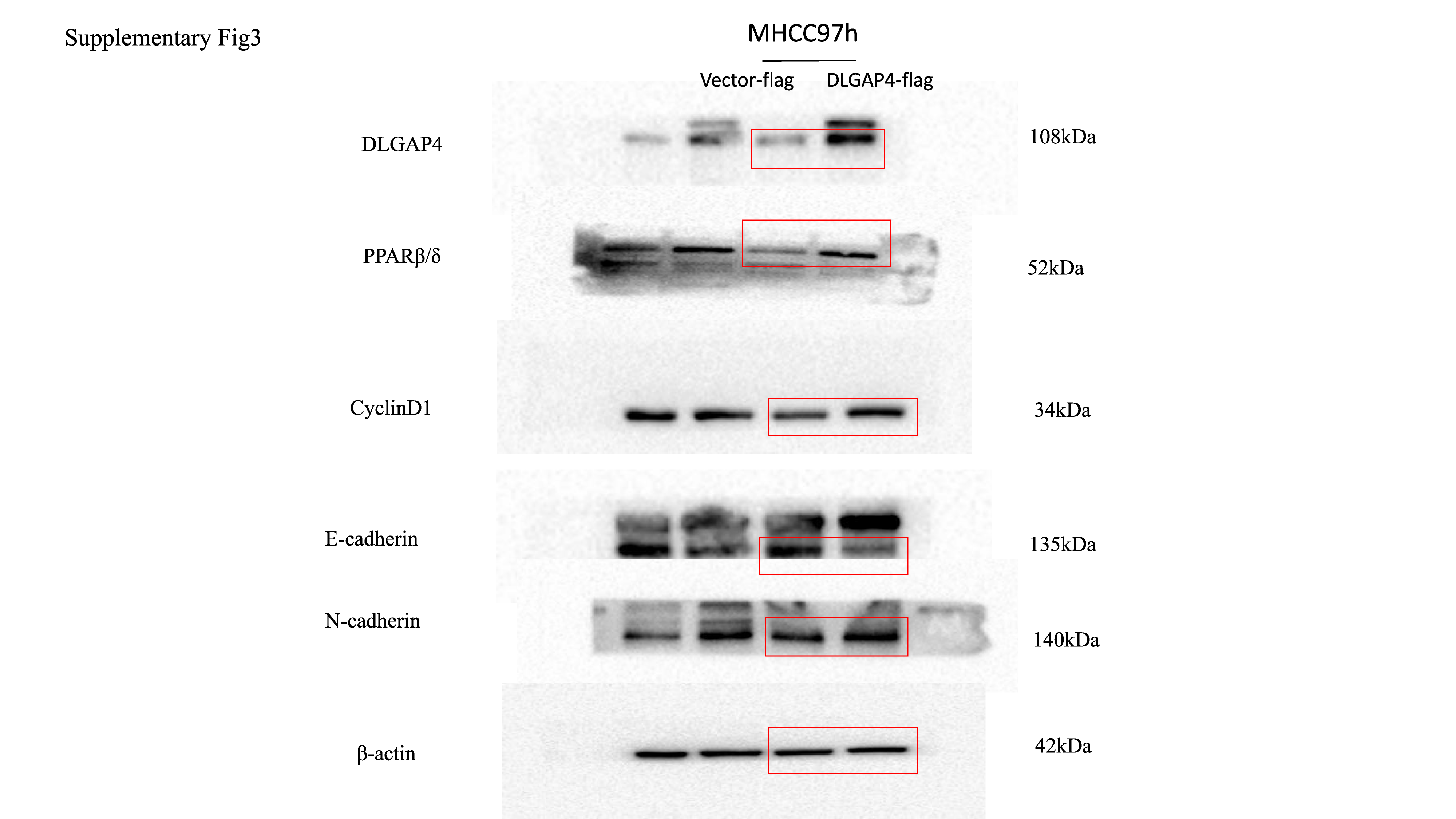

Supplement: Supplementary file 7 — Supplementary Information 7. [file 41598_2022_23837_MOESM7_ESM.tif]
